# CRABP-II- and FABP5-independent responsiveness of human glioblastoma cells to all-trans retinoic acid

**DOI:** 10.18632/oncotarget.3334

**Published:** 2015-01-21

**Authors:** Shi-Lin Xia, Mo-Li Wu, Hong Li, Jia-Hui Wang, Nan-Nan Chen, Xiao-Yan Chen, Qing-You Kong, Zheng Sun, Jia Liu

**Affiliations:** ^1^ Liaoning Laboratory of Cancer Genetics and Epigenetics and Department of Cell Biology, College of Basic Medical Sciences, Dalian Medical University, Dalian, China; ^2^ Department of Hematology, PLA 210 Hospital, Dalian, China

**Keywords:** Glioblastoma, All-trans retinoic acid, Chemosensitivity, CRABP-II, FABP5

## Abstract

Glioblastomas respond differently to all-trans retinoic acid (RA) for unknown reasons. Because CRABP-II and FABP5 mediate RA intracellular signaling respectively and lead to distinct biological consequences, their expression patterns in different grades of astrocytomas and the glioblastoma cells lines LN18, LN428 and U251 were examined to identify potential correlations with RA sensitivities. The response of glioblastoma cells to RA, decitabine or the FABP5 competitive inhibitor, BMS309403, was analyzed. CRABP-II and FABP5 were expressed to varying degrees by the 84-astrocytoma cases examined. Treatment of LN428, U251 and LN18 cells with RA failed to suppress their growth; however, U251 proliferation was inhibited by decitabine. The combination of decitabine and RA suppressed the growth of all three cell lines and induced significant apoptosis of LN428 and U251 cells. Both CRABP-II and FABP5 were transcribed in the three cell lines but FABP5 proteins were undetectable in U251 cells. The ratio of CRABP-II to FABP5 was not altered after RA, decitabine or RA and decitabine treatment and the resistance of cells to RA was not reversed by BMS309403 treatment. In conclusion, CRABP-II and FABP5 expression patterns are neither related to the tumor grades nor correlated with RA sensitivity. Additional molecular factors may be present that determines the sensitivity of glioblastoma cells to RA. Dicitabine may improve the sensitivity of glioblastoma cells to RA, however, its underlying mechanism and its *in vivo* feasibility need to be investigated.

## INTRODUCTION

Glioblastoma multiforme (GBM) is the most aggressive and common brain malignancy in adults [1]. Because of the difficulty in removing the tumor radically, the therapeutic outcome of GBM is very poor. Current treatment consists of surgery with radiotherapy and/or chemotherapy, however, the average survival of GBM patients is only 12-15 months [2-5]. Evidence indicates that GBM responds differently to anticancer drugs and intensive adjuvant treatments usually lead to local and systemic damage to the patients [6-8]. Understanding the mechanisms of GBM cell chemoresistance would be of significant clinical value.

As a natural derivative of vitamin A, all-trans retinoic acid (RA) regulates multiple biological processes, including growth suppression of certain normal and malignant cells [9-12]. RA is able to inhibit the growth of promyelocytes and medulloblastoma cells by promoting their differentiation and apoptosis [13, 14]. However, in other cancers, RA appears to promote rather than inhibit cell survival. For example, RA can promote cell survival and growth of NaF mammary tumor cells of the MMTV-neu mouse [15] and enhance skin tumor formation [16]. The data concerning the biological effects of RA on human glioblastoma cells are controversial. Some studies have demonstrated a therapeutic benefit of RA in the treatment of human glioblastomas [17], whereas, other studies found that retinoids failed to inhibit proliferation and migration of human glioblastoma cells [18]. In addition, RA has been demonstrated to enhance the transcription of a group of cancer-associated genes in glioblastoma cells [19]. Nevertheless, the cause of different RA sensitivities in glioblastoma cells is unknown.

RA can signal via two classical pathways, mediated by CRABP-II or FABP5, which result in distinct cellular responses [15]. CRABP-II delivers RA from the cytosol to nuclear retinoic acid receptors (RARs), which associate with retinoid X receptor (RXRs) to form heterodimers. These complexes bind to regulatory regions of specific target genes, leading to differentiation, cell-cycle arrest and apoptosis [9-12, 15]. Conversely, the RA signal mediated by the FABP5/PPAR pathway may promote cancer cell survival by activating some cancer-associated genes [19, 20]. An imbalanced ratio of CRABP-II and FABP5 may determine RA sensitivities of cancer cells [15]. This notion is supported by research in human medulloblastoma cells, as the RA-resistance of CRABP-II-negative UW228-2 and UW228-3 medulloblastoma cells can be reversed via restoration of CRABP-II expression. In addition, Med-3 cells become RA-resistant when their CRABP-II expression is specifically blocked [13]. Because glioblastoma cells also show different RA sensitivities [17, 18, 21-23], it is possible that their response to RA may be similar to that of medulloblastoma cells. To determine this, the statuses of CRABP-II and FABP5 mediated-RA signaling and their correlations with RA sensitivities were investigated using three glioblastoma cell lines.

## RESULTS

### Differential CRABP-II and FABP5 expression patterns of astrocytomas

**Figure 1:**
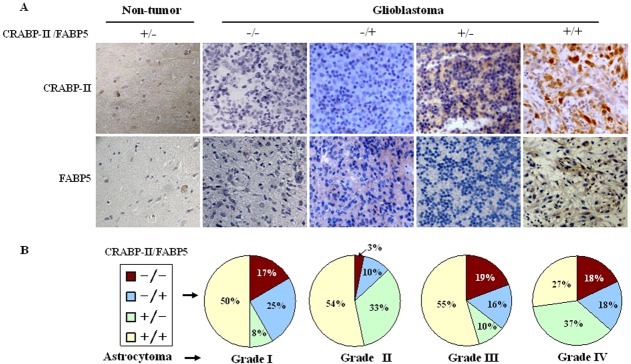
Expression patterns of CRABP-II and FABP5 in non-tumor brain tissues and astrocytomas The staining patterns were scored as “−” if no immunolabeling was observed in the cells and “+” if distinct staining was generally observed. (A) Immunohistochemical profiling of CRABP-II and FABP5 expression patterns in non-tumor brain tissues and glioblastomas. (B) The percentage of CRABP-II and FABP5 expression in the four astrocytoma grades.

A total of 84 astrocytoma specimens were classified into Grade I, II, III or IV according to the criteria of World Health Organization classification system [1] (Table 1). The expression patterns of CRABP-II and FABP5 in the four grades of astrocytomas were analyzed using tissue microarray-based immunohistochemical staining. CRABP II and FABP5 were expressed differently within the four-astrocytoma grades (Table 1). CRABP II and FABP5 were expressed, respectively, in 58.3% (7/12) and 75% (9/12) of grade I astrocytomas, 86.7% (26/30) and 63.3% (19/30) of Grade II, 64.5% (20/31) and 71% (22/31) of Grade III, and 63.6% (7/11) and 45.5% (5/11) in Grade IV. The staining patterns or labeling densities of CRABP-II and FABP5 in the four grades of astrocytomas were analyzed and compared with their surrounding noncancerous counterparts (Figure 1A). The surrounding tissues were positive for CRABP-II but negative for FABP5 (Figure 1A). The four staining patterns of CRABP-II and FABP5 were observed among glioblastomas: CRABP-II^−^/FABP5^−^, CRABP-II^−^/FABP5^+^, CRABP-II^+^/FABP5^−^ and CRABP-II^+^/FABP5^+^ and the respective profiles were expressed by 18% (2/11), 18% (2/11), 37% (4/11) and 27% (3/11) of the glioblastomas (Figure 1B).

**Table 1:** the expression patterns of CRABP-II and FABP5 in different grades of human astrocytomas

	CRABP-II					FABP5				
Astrocytoma Grades	Positive rate (%)	Expression levels (case number)				Positive rate (%)	Expression levels (case number)			
		−	+	++	+++		−	+	++	+++
I	7/12 (58.3)	5	0	7	0	9/12 (75)	3	4	5	0
II	26/30 (86.7)	4	15	10	1	19/30 (63.3)	11	8	10	1
III	20/31 (64.5)	11	11	5	4	22/31 (71)	9	11	9	2
IV	7/11 (63.6)	4	4	2	1	5/11 (45.5)	6	2	2	1

### RA-resistant properties of LN18, LN428 and U251 cells

**Figure 2:**
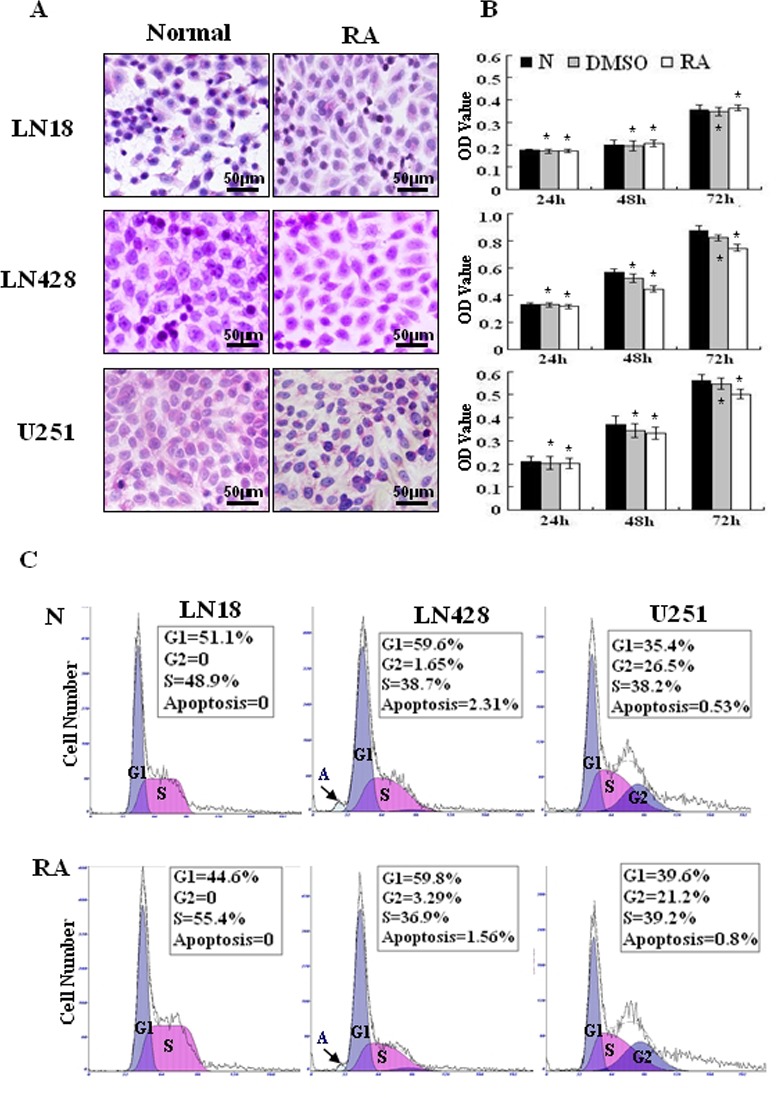
Cellular responses of human glioblastoma LN18, LN428 and U251 cells to all-trans retinoic acid treatment (A) Hematoxylin and eosin morphological staining was performed on LN18, LN428 and U251 cells that were either untreated (N) or treated with 10 μM of RA for 48 h (RA). (B) Evaluation of the responses of LN18, LN428 and U251 to 10 μM of RA for 72 h using a MTT cell proliferation assay, compared with the untreated group (N), *p>0.05. (C) Flow cytometry performed on LN18, LN428 and U251 cells under normal culture conditions and after incubation with 10 μM of RA for 48 h.

The effects of RA on the three human glioblastoma cells were evaluated by continuously treating them with 10 μM of RA for 72 h. Hematoxylin and eosin staining revealed no obvious morphological changes among the RA-treated cells in comparison with the normally cultured or 0.2% DMSO treated cells (Figure 2A). An MTT assay (Figure 2B) on RA treated LN428 cells indicated a 20.9% reduced cell viability compared to untreated cells and a 14% reduction compared with DMSO treated cells. U251 cells treated with RA for 48 h displayed a 10.8% reduced cell viability compared with untreated cells and 4.2% compared with DMSO treated cells. No statistical significance was identified for LN428 (*p*=0.093) or for U251 cells (*p*=0.18). In comparison with the number of cells in the untreated LN18 cells, 0.2% DMSO reduced the population by 4.2% (*p*=0.118) and 48 h of RA treatment increased the population by 2.6%. Flow cytometry analysis demonstrated that the cell cycle fractions of LN18, LN428 and U251 cells were similar when untreated or treated with 10 μM of RA. Furthermore, apoptosis was infrequent in each of the experimental groups (Figure 2C).

### Detection of CRABP-II in the three-glioblastoma cell lines

**Figure 3:**
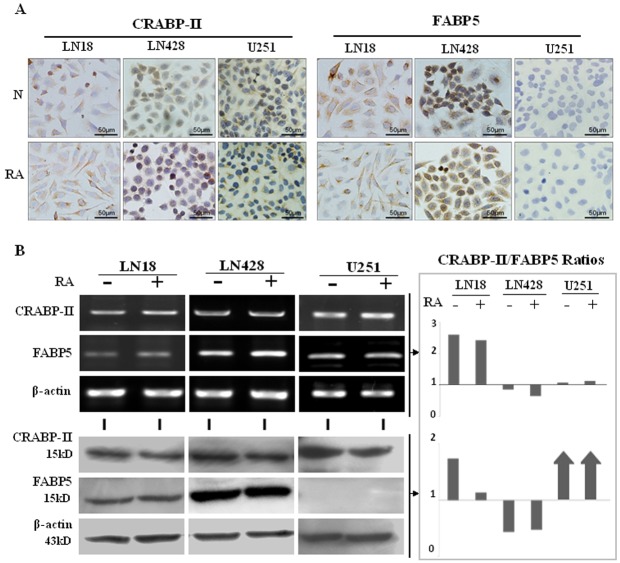
CRABP-II and FABP5 expression patterns in LN18, LN428 and U251 cells with and without RA treatment (A) Immunocytochemical staining of CRABP-II and FABP5 expression in the three cell lines that were either untreated (N) or treated with RA (RA). (B) RT-PCR and western blot analyses of CRABP-II and FABP5 expression in LN18, LN428 and U251 cells with and without RA treatment. CRABP-II/FABP5 ratios were calculated according to the results of RT-PCR and western blotting.

Immunocytochemical staining showed that the levels of CRABP-II expression in LN18, LN428 and U251 cells were almost identical between the untreated and the RA-treated cells (Figure 3A). Accordingly, RT-PCR and western blot analyses revealed that CRABP-II was expressed in LN18, LN428 and U251 cells and its levels remained unchanged following RA treatment (Figure 3B).

### Differential FABP5 expression patterns in the three-glioblastoma cells

According to the results of RT-PCR, ICC and western blot analyses, FABP5 was expressed in LN18 and LN428 cells and the levels were not changed after RA treatment. FABP5 RT-PCR products could be generated in low levels from U251 cell RNA, in the presence or absence of RA treatment, but the FABP5 protein was undetectable (Figure 3A and 3B). These results are supported by a previous study [24]. DNA sequencing confirmed that the 281 bp RT-PCR product was amplified with FABP5 primers and was specifically generated from FABP5 transcripts, which encompassed Exon 1 to Exon 3 (data not shown).

### Variable CRABP-II and FABP5 ratios among glioblastoma cell lines

Based on the RT-PCR and western blot results, the ratios of CRABP-II and FABP5 were calculated at the transcriptional and translational levels. It was found that the ratio of CRABP-II to FABP5 transcripts were 2.6 in LN18, 0.85 in LN428 and 1.08 in U251 cells, which showed little change (2.43, 0.64 and 1.13, respectively) after RA treatment (Figure 3B). The ratio of CRABP-II to FABP5 proteins were 1.17 and 0.74 in untreated LN18 and LN428 cells, respectively, and 1.03 and 0.65, respectively, following RA treatment. Because FABP5 protein was not detected in U251 cells, CRABP-II was the predominant protein.

### RA up-regulated CYP26A1 expression

**Figure 4:**
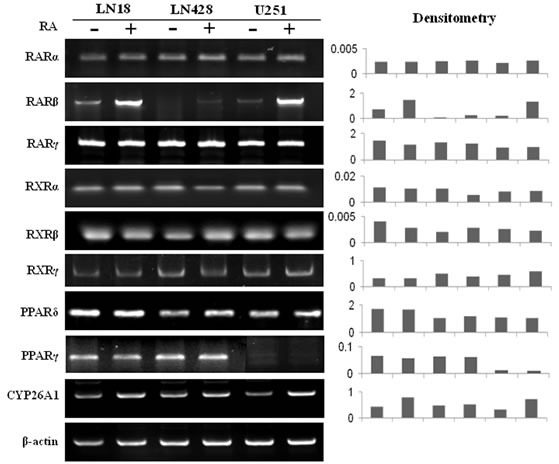
RT-PCR profiling of the expression of RARs, RXRs, PPARδ, PPARγ and CYP26A1 in LN18, LN428 and U251 cells before and after RA treatment

The catabolic enzyme CYP26A1, which is essential for RA catabolism, is known to promote RA degradation and extracellular elimination [25]. The results of RT-PCR revealed that expression of CYP26A1 was up-regulated following RA treatment (Figure 4). This indicates that the cellular response to exogenous RA is to increase CYP26A1 expression and thereby induce RA catabolism.

### Differential expression of RA nuclear receptors

Because of the importance of the nuclear RA receptors, PPARδ and PPARγ, in mediating RA target gene transcription, their expression patterns in the three cell lines were examined before and after RA treatment. RT-PCR revealed that RARs, RXRs and PPARδ were expressed in all three cell lines, although the level of RARβ expression was low, especially in LN428 cells (Figure 4). In RA-treated LN18 and U251 cells, RARα, RARγ, RXRs and PPARδ expression levels remained stable, while RARβ was clearly up-regulated. In the case of LN428 cells, RA treatment slightly increased RARβ expression, while the levels of PPARδ and other RA nuclear receptors were unchanged. PPARγ expression in LN18 and LN428 cells was unchanged after RA treatment, but was slightly down-regulated in U251 cells after RA treatment.

### Decitabine improved the RA sensitivity of glioblastoma cells

**Figure 5:**
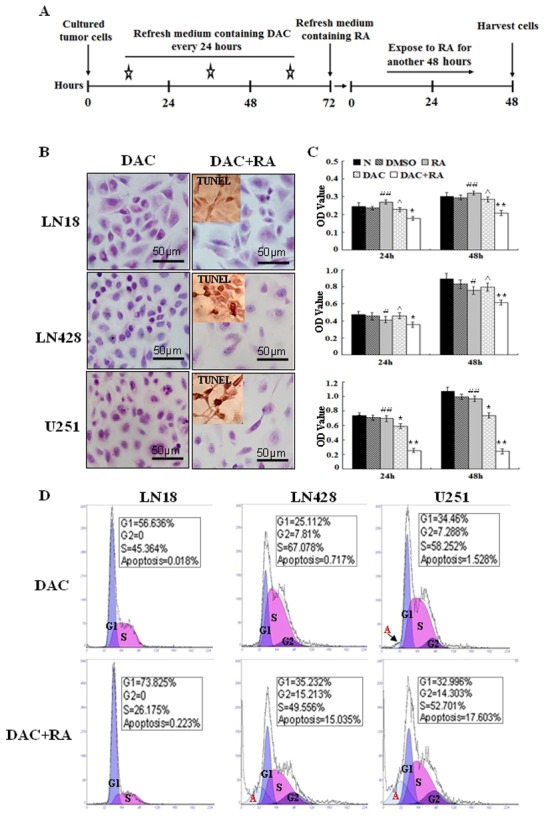
The effect of decitabine and its combination with retinoic acid on the growth and survival of glioblastoma cells (A) A schematic diagram of glioblastoma cells treated with decitabine and decitabine in combination with retinoic acid. (B) Morphological features of LN18, LN428 and U251 cells after decitabine (DAC) and combinational treatment (DAC+RA). The insets, The images of TUNEL apoptosis assay (C) An MTT assay performed on LN18, LN428 and U251 cells that were untreated (N), treated with DMSO, 10 μM of RA for 48 h (RA), 1 μM decitabine for 72 h (DAC) or 10 μM of RA for 48 h after pre-treated for 72 h with 1 μM decitabine (DAC+RA). *, compared with N group, *p*<0.05; **, compared with N group, *p*<0.01; #, compared with DAC+RA, *p*<0.05; ##, compared with DAC+RA, *p*<0.01; ^, compared with N group, *p*>0.05. (D) Flow cytometry was performed on LN18, LN428 and U251 cells after culture with 1 μM of decitabine for 72 h (DAC) and after incubation with 1 μM decitabine for 72 h followed by 10 μM RA for 48 h (DAC+RA).

After treatment with 1 μM of decitabine for 72 h, the three cell lines were cultured with or without RA for another 48 h (Figure 5A) [26]. The results revealed that decitabine directly suppressed proliferation of U251 cells by 30.7%, but it had little effect on LN18 and LN428 cells (Figure 5B and 5C). The effect of the combined decitabine and RA treatment on the three cell lines varied. The combined treatment for 48 h could suppress the growth of LN18, LN428 and U251 cells by 31.1% (*p*=0.001), 32.9% (*p*=0.002) and 77.1% (*p*=0.001), respectively (Figure 5C). Flow cytometry analyses showed that decitabine and RA combined treatment led to increased apoptosis of LN428 by 15% and U251 cells by 17.6%. Although apoptosis was not increased in LN18 cells, a significant proportion were arrested in the G1 phase (73.8%), while cells in the G2 phase were undetectable (Figure 5D). In addition, a TUNEL assay identified frequent apoptotic death in LN428 and U251 cells treated with decitabine and RA (Figure 5B).

### Differential responses of RA-related elements to decitabine

**Figure 6:**
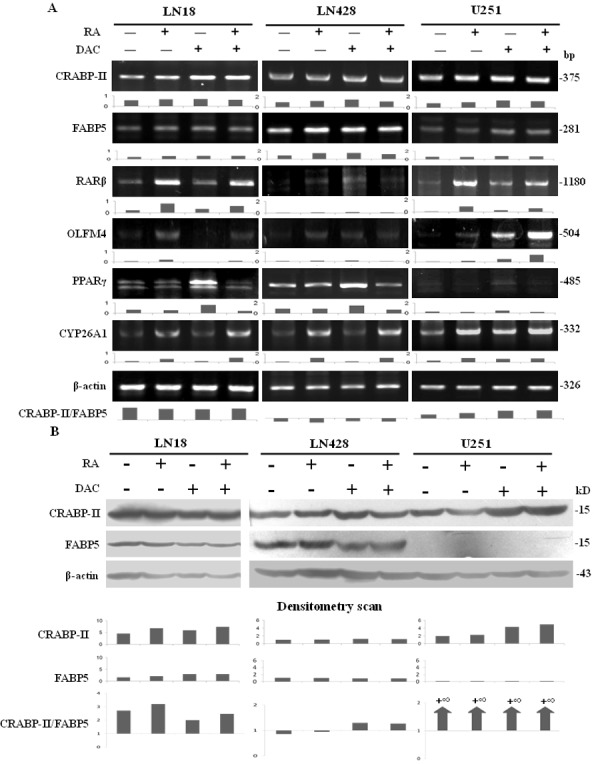
The responses of CRABP-II- and FABP5-related elements to retinoic acid, decitabine and their combined treatment (A) RT-PCR examination of CRABP-II, FABP5, RARβ, OLFM4, PPARγ and CYP26A1 transcription under the following conditions: normal culture, 10 μM of RA for 48 h, 1 μM decitabine for 72 h and 10 μM of RA for 48 h after pre-treatment with 1 μM decitabine for 72 h. CRABP-II/FABP5 ratios were calculated according to RT-PCR data. (B) CRABP-II and FABP5 western blotting performed on LN18, LN428 and U251 cells under the following conditions: normal culture, 10 μM of RA for 48 h and 1 μM decitabine pretreatment for 72 h followed by 10 μM of RA for 48 h. CRABP-II/FABP5 ratios were calculated according to the data obtained.

RT-PCR and western blotting showed that neither CRABP-II nor FABP5 was up-regulated in LN18 and LN428 cells after decitabine or decitabine and RA treatment (Figure 6A and 6B). In the case of U251 cells, CRABP-II expression was relatively stable, while FABP5 was only detectable in the transcriptional rather than translational level after decitabine or decitabine and RA treatment (Figure 6B). Consequently, the CRABP-II/FABP5 ratios in three cell lines were not apparently altered after RA, decitabine or their combined treatments (Figure 6A and 6B). The expression level of RARβ remained stable after decitabine treatment and was up-regulated with the combined treatment in LN18 and U251 cells. RARβ expression was increased in LN428 cells after decitabine treatment and decitabine and RA combined treatment. PPARγ expression was up-regulated in LN18, LN428 and U251 cells after decitabine treatment, but was almost unchanged after decitabine and RA treatment. The expression of CYP26A1 remained unchanged after decitabine treatment but was up-regulated after decitabine and RA treatment in LN18 and LN428 cells. CYP26A1 expression was increased in U251 cells treated with decitabine and the combined treatment (Figure 6A). OLFM4 is known as a target of RA and the activation of OLFM4 may contribute to the therapeutic effect of RA [27]. The expression of OLFM4 in the three cell lines, with or without RA treatment, was therefore examined by RT-PCR. OLFM4 was up-regulation in LN18 and U251 cells by both RA treatment and decitabine and RA combined treatment, but only extremely low levels were detected in LN428 cells irrespective of the treatment (Figure 6A).

### Failure to reverse RA resistance by FABP5 inhibition

**Figure 7:**
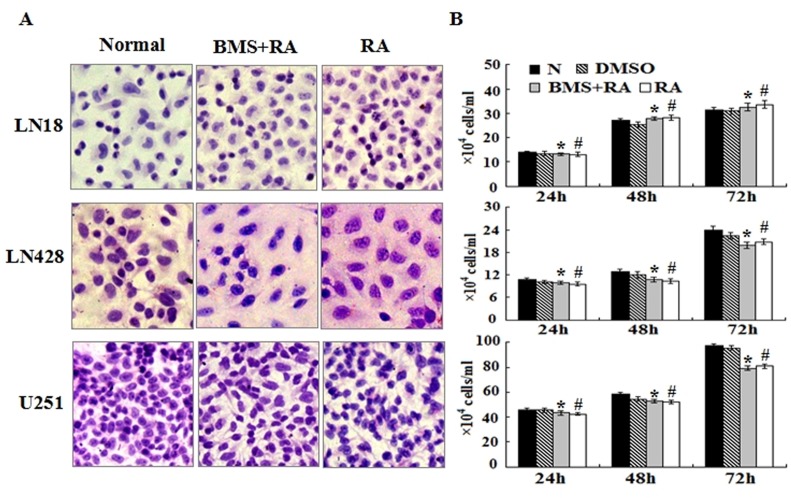
The effect of the FABP5 inhibitor, BMS309403, on the growth and survival of glioblastoma cells treated with RA (A) Hematoxylin and eosin morphological staining performed on LN18, LN428 and U251 cells under the following conditions: normal culture; 25 μM BMS309403 pretreatment for 6 h followed by 10 μM RA treatment for 48 h (BMS+RA) and 10 μM RA treatment for 48 h (RA). (B) Evaluation of the responses by cell counting of LN18, LN428 and U251 treated with 25 μM of BMS309403 for 6 h before 10 μM of RA and 10 μM RA for 72 h. *, compared with N group, *p*>0.05; #, compared with BMS+RA group, *p*>0.05.

To determine the effect of FABP5 inhibition on the RA resistant features of LN18, LN428 and U251 cells, BMS309403, a competitive inhibitor of FABP5 [28, 29], was used to treat glioblastoma cells prior to RA treatment. Hematoxylin and eosin staining revealed no obvious morphological changes among the cells pretreated for 6 h with BMS309403 before RA treatment, when compared to untreated cells and cells treated with RA only (Figure 7A). The results from cell counting showed similar growth speeds between the RA-treated glioblastoma cells and the cells treated with BMS309403 before RA treatment (Figure 7B). In addition, the percent of viable cells after 72 h of culture in the untreated, RA treated and BMS309403 and RA treated groups were 97%, 96.8% and 96%, respectively, for LN18 cells; 98%, 97.2% and 97.8%, respectively, for LN428 cells; 98%, 96% and 96.2%, respectively, for U251 cells. These results suggest that BMS309403 failed to reverse RA resistance among glioblastoma cells.

## DISCUSSION

The effect of RA on human glioblastomas has been intensively investigated, with the aim of finding a method to improve the therapeutic outcome of this lethal malignancy [17, 18, 21-23]. However, the data obtained are controversial. Some reports demonstrate that RA exhibits a heterogeneous growth-inhibitory activity against human glioblastoma cells by modulation of EGF receptor phosphotyrosine kinase activity [30, 31]. However, a body of evidence has revealed frequent RA resistance among glioblastoma tumors and cell lines [18, 21, 22]. As demonstrated in the current study, the three cell lines examined were resistant (LN18) or insensitive (LN428 and U251) to RA, and treatment caused little morphological alternation, very low apoptosis frequencies and unchanged growth rates. Moreover, RA-treated LN18 cells grew more rapidly. Given these data, it is necessary to explore the molecular mechanism underlying the different RA sensitivities to enable reliable application of RA for the treatment of glioblastomas.

There are two well-characterized RA signal transduction pathways that are mediated by CRABP-II and by FABP5. Because the biological consequences of these two pathways are distinct, the expression levels of CRABP-II and FABP5 may determine the RA sensitivities and outcome of cancer cells treated with RA [15]. For example, it was reported that the expression ratio of FABP5 to CRABP-II was significantly higher in the tumor tissues of short-term survivors compared to long-term survivors. In addition, the expression of FABP5 protein was increased in highly proliferating tumor cells [21]. For this reason, CRABP-II and FABP5 expression patterns and their ratios in different grades of astrocytomas were profiled immunohistochemically. It was revealed that CRABP-II and FABP5 were expressed in four forms as CRABP-II^−^/FABP5^−^, CRABP-II^−^/FABP5^+^, CRABP-II^+^/FABP5^−^ and CRABP-II^+^/FABP5^+^ in the different grades of astrocytomas and the expression was unrelated to the tumor grade. According to the proposed functions of the two classical RA signaling pathways [15] and our data from human medulloblastoma cells [13], it was possible that the distinct CRABP-II and FABP5 expression patterns found randomly in the four grades of astrocytomas might explain the variable RA sensitivities of individual glioblastoma cases. This hypothesis was therefore tested using LN-18, LN-428 and U251 cell lines.

The *in vitro* results showed that CRABP-II was expressed in all three cell lines used and its levels remained stable after RA treatment. FABP5 could be detected at both RNA and protein levels in LN18 and LN428 cells, while it was transcribed to mRNA but not translated to protein in U251 cells. Consequently, the CRABP-II and FABP5 ratios of the three RA-insensitive cell lines were not identical. According to the findings from other types of cancers, the cells with high CRABP-II expression tend to be sensitive to RA treatment and cells with high FABP5 expression tend to be insensitive to RA treatment [9-12, 13, 32]. However, the results from this study contradict these findings, as CRABP-II protein expression was 2.6-fold higher than FABP5 in RA resistant LN18 cells and the RA-insensitive U251 cells expressed CRABP-II in the absence of FABP5. Because the major components of CRABP-II and FABP5 expression did not correlate with RA sensitivity, it is possible that CRABP-II and FABP5 are not the critical determinants of RA sensitivities in glioblastoma cells. Therefore, our results suggest the presence of additional mechanism related to the response of glioblastoma cells to RA treatment.

Gene expression patterns can be modified by epigenetic alterations, including DNA methylation. Decitabine, a powerful DNA methylation eraser, has therefore been increasingly used independently or in combination with other agents in cancer therapy [26]. RA and decitabine combination has been employed to treat patients with leukemia, breast and thyroid cancers [27, 33, 34]. This treatment method can restore key RA signaling pathways that enable cancer cells to become sensitive to RA [13]. This strategy has been attempted on human glioblastomas as well. One study found that 5-aza-2′-deoxycytidine-treated primary cultured glioblastoma cells showed CRABP-II up-regulation, which rendered them more sensitive to RA treatment [22]. In the current study, a similar treatment regimen was used to determine whether the RA-resistant properties of the three glioblastoma cell lines could be overcome. Although decitabine exerted little effect on CRABP-II and FABP5 expression patterns, it enhanced RA sensitivities of CRABP-II^++^/FABP5^++^ LN428 cells and CRABP-II^++^/FABP5^−^ U251 cells in terms of growth arrest and apoptosis. However, CRABP-II^++^/FABP5^+^ LN18 cells were less sensitive to RA and decitabine treatment, although significantly more cells were in the G1 phase. These phenomena suggest that CRABP-II and FABP5 levels or ratios are not associated with the responsiveness of human glioblastoma cells to RA. The failure of the three glioblastoma cells to overcome RA resistance with the FABP5 competitive inhibitor, BMS309403, further supports this notion. Our findings using human glioblastoma cells contradict the currently working hypothesis on RA sensitivity [13, 15] and indicate that the molecular mechanisms of RA intracellular signaling and the cellular responses to RA are more complicated than expected.

Current data has revealed that additional factors are involved in regulation of RA sensitivity beyond the classical pathways mediated by CRABP-II and by FABP5 [35]. OLFM4 is known as a novel target of RA and the activation of OLFM4 may contribute to the therapeutic value of RA [27]. However, the biological effects of OLFM4 differ between the types of cancer. For instance, it functions as an anti-apoptotic factor in pancreatic cells by promoting S-phase transition [36] and it also promotes metastasis of colon cancers [37]. In contrast, OLFM4 overexpression leads HL-60 cells to growth inhibition, differentiation and apoptosis [27]. Currently, the expression pattern and the biological roles of OLFM4 in glioblastoma cells remain unknown. Our results clearly reveal that the levels of OLFM4 expression are extremely low in LN18, LN428 and U251 cells but are up-regulated after RA and decitabine and RA treatments. Interestingly, RA can up-regulate OLFM4 expression 3.38, 1.25 and 2.37 fold in RA-resistant LN18 cells and RA-insensitive LN428 and U251 cells, respectively. These findings demonstrate for the first time that RA promotes OLFM4 expression and suggests a potential favorable role of OLFM4 in human glioblastoma cells.

Taken together, the current study demonstrates variable expression patterns of CRABP-II and FABP5 among the four astrocytoma grades. LN18, LN428 and U251 glioblastoma cell lines are insensitive to RA treatment irrespective to their CRABP-II and FABP5 expression statuses or FABP5 inhibition. The global DNA demethylator decitabine can overcome RA resistance or insensitivity of the three cell lines without altering the levels of CRABP-II and FABP5 expression. OLFM4 proteins might play anti-apoptotic roles in RA-treated glioblastoma cells but are not sufficient to rescue decitabine-pretreated LN428 and U251 cells from RA-induced apoptosis. Because neither the CRABP-II- nor the FABP5-mediated RA signaling pathway is the key determinant of RA sensitivity in glioblastoma cells, it would be worthwhile to search for other RA-related factors that indicate RA sensitivity to enable reliable anti-glioblastoma therapy. In addition, it would be necessary to explore alternative treatments for glioblastoma to provide greater efficacy and less toxicity because RA and decitabine combination has uncertain *in vivo* feasibility, safety and efficacy and because of the frequent resistance of glioblastoma cells to RA.

## MATERIALS AND METHODS

### Tissue microarray-based immunohistochemical (IHC) staining

84 paraffin-embedded astrocytoma specimens with various grades were collected from the First Affiliated Hospital of Dalian Medical University. This study was approved by the hospital institution review board and informed consent was obtained from all patients. The expression patterns of CRABP-II and FABP5 in different grades of astrocytomas were profiled immunohistochemically, using paraffin sections of tissue microarrays constructed in duplicate with 84 astrocytomas and, where possible, noncancerous tumor surrounding tissues [38]. The antibodies used were rabbit anti-human CRABP II and FABP5 (Proteintech, Chicago, IL, USA) at dilutions of 1:100 and 1:80, respectively. Color reaction was developed using 3, 3′-diaminobenzidine tetrahydrochloride (DAB). The sections without the first antibody incubation were used as the background control. According to the labeling intensity, the staining results were evaluated by two researchers, and scored as negative (−) if no immunolabeling was observed in target cells, weakly positive (+) if the labeling was faint, moderately positive (++) if the labeling was stronger, and strongly positive (+++) if the labeling was distinctly stronger than (++) [39].

### Cell culture and treatments

Human glioblastoma LN18 and LN428 cell lines were kindly provided by Professor Nicolas de Tribolet, Department of Neurosurgery, Central Hospital University of Laussane, Switserland and human glioblastoma U251 cell line was obtained from the Cell Culture Facility, Chinese Academy of Sciences Cell Bank, Shanghai. The cells were cultured in DMEM (Gibco, USA) supplemented with 10% fetal bovine serum (Gibco, USA) under 37°C and 5% CO_2_ condition and were plated onto culture dishes (Nunc A/S, Roskilde, Denmark) at a density of 5×10^4^/ml, and incubated for 24h before further experiments. For H/E, ICC staining and TUNEL assay (Promega, Madison, WI, USA), dozens of cell-bearing coverslips were concurrently prepared using the Nest-Dishes (Nest Biotech. Inc., Wuxi, China; China invention patent No. ZL200610047607.8) and collected regularly during drug treatments.

All-trans retinoic acid (RA; Sigma-Aldrich, St. Louis, USA) was dissolved in dimethylsulfoxide (DMSO; Sigma-Aldrich) and diluted with culture medium to the optimal working concentration (10μM) [13] just before use. The cells were treated by 10μM RA for 72h, meanwhile the cells under normal culture condition and treated by 0.2% DMSO were used as normal and background controls, respectively. Cell numbers and viabilities were checked in 12 h intervals and the cell-bearing coverslips were fixed in cold acetone or 4% paraformaldehyde (pH 7.4) for morphological, immunocytochemical examinations and TUNEL assay. The experimental groups were set in triplicate and the experiments were repeated for three times to establish confidential conclusion.

### Cell proliferation and death assays

The effects of RA, DAC and DAC/RA combination on cell proliferation were determined by 3-[4,5-Dimethylthiazol-2-yl]-2,5-diphenyl-tetrazolium bromide/MTT assay [40]. The results were shown as percentage of cell viability (OD of the experiment samples/OD of the control) or OD values. Terminal deoxynucleotide transferase (TdT)-mediated dUTP-biotin nick-end labeling (TUNEL) assay was performed on the cell-bearing coverslips to detect apoptotic cells according to producer's instructions (Promega Corporation, USA).

### Flow cytometry analysis

The harvested cells of the experimental groups were fixed in ethanol for staining with DNA dye and re-suspended in 0.5 ml to 1 ml propidium iodide (PI) solution containing RNase and incubated at 37°C for 30 min. Cell cycle profiles and the proportion of apoptotic cells were determined with a FACS vantage Flow Cytometer (Becton Dickinson, San Jose, CA, USA), and the data were analyzed with MOD FIT software (Verity Software House, Inc, Topsham, ME, USA).

### RNA isolation and RT-PCR

Total cellular RNAs of the experimental groups were extracted using Trizol solution (Life Technology, Grand Island, NY, USA). The sample RNAs were subjected to reverse transcription/RT and then polymerase chain reaction/PCR using the primers specific for CRABP-II, RARα, RARβ, RARγ, RXRα, RXRβ, RXRγ, FABP5, PPARδ, PPARγ, CYP26A1 and OLFM4 according to producer's protocols (Takara Inc., Dalian Branch, Dalian, China). The sequences of PCR primers for each of the gene transcripts were listed in Table 2. The PCR products were resolved on ethidium bromide-stained 1.5% agarose gel and photographed under UV illumination (UVP, LLC, Upland, CA, USA). β-actin products generated from the same RT solutions were used as quantitative control.

**Table 2:** Primer sequences, amplicon size and annealing temperature for RT-PCR

Gene	Primers	Amplicon Size (bp)	Annealing °C
CRABP-II	F: 5′-ATGCCCAACTTCTCTGGCAA-3′ R: 5′-CGTCATGGTCAGGATCAGTT-3′	375	59
FABP5	F: 5′-AGCAGCTGGAAGGAAGATGG-3′ R: 5′-CTGATGCTGAACCAATGCAC-3′	281	55
RARα	F:5′-GTGTCACCGGGACAAGAACT-3′ R:5′-CGTCAGCGTGTAGCTCTCAG-3′	175	66
RARβ	F:5′-GAATTGAAACACAGAGCACC-3′ R:5′-GCAGGAGTGGTGACTGACTG-3′	1180	54
RARγ	F:5′-CCACCAATAAGGAGCGACTCTTTG-3′ R:5′-TTCTTCTGGATGCTTCGGCG-3′	358	55
RXRα	F:5′-CCCTGTCACCAACATTTGC-3′ R:5′-AGAAGTGTGGGATCCGCTTG-3′	90	60
RXRβ	F:5′-CTCTGGATGATCAGGTCATATTGCT-3′ R:5′-GCCATCTCG AACATCAATGGA-3′	92	60
RXRγ	F:5′-GGGAAGCTGTGCAAGAAGAAA-3′ R:5′-TGGTAGCACATTCTGCCTCACT-3′	69	60
PPARδ	F: 5′-CAGAAGAAGAACCGCAACA-3′ R: 5′-CGCCATACTTGAGAAGGGT-3′	503	50
PPARγ	F: 5′-TATCATAAATAAGCTTCAATCG-3′ R: 5′-GAGATGGAATTCTGGCCCACC-3′	485	60
CYP26A1	F:5′-GAGACCCTTCGACTGAATCC-3′ R:5′-GGAGGTCCATTTAGAAGCTGC-3′	332	56
OLFM4	F:5′-TATGGTGCTTGGGGTAGGGA-3′ R:5′-CCCACATACCATGAAGGCGT-3′	504	55
β-actin	F:5′-GCATGGAGTCCTGTGGCAT-3′ R:5′-CTAGAAGCATTTGCGGTGG-3′	326	58

### Immunocytochemical staining

CRABP II and FABP5 immunocytochemical (ICC) staining were performed on the coverslips collected from different experimental groups with the method described elsewhere [40]. The antibodies used were rabbit anti-human CRABP II and rabbit anti-human FABP5 (Proteintech, Chicago, IL, USA) at dilutions of 1:100 and 1:80, respectively. The sections without the first antibody incubation were used as the background control.

### Protein preparation and Western blotting

Total cellular proteins were prepared from the cells under different culture conditions [41]. The sample proteins (15μg/well) were separated by electrophoresis in 10% sodium dodecylsulfate–polyacrylamide gel electrophoresis and transferred to polyvinylidene difluoride membrane (Amersham, Buckinghamshire, UK). The membrane was blocked with 5% skimmed milk in TBS-T (10 mM Tris–Cl, pH 8.0, 150 mM NaCl and 0.5% Tween 20) at 4°C overnight, rinsed three times with TBS-T and followed by 3h incubation at room temperature with the first antibody, and 1h incubation with HRP-conjugated anti-rabbit IgG (Zymed Lab Inc., San Francisco, CA, USA). The bound antibody was detected using the enhanced chemiluminescence system (Roche, Penzberg, Germany). After removing the labeling signal by incubation with stripping buffer (62.5 mM Tris–HCl, pH 6.7, 100 mM 2-mercaptoethanol, 2% SDS) at 55°C for 30 min, the membrane was reprobed with other antibodies one-by-one until all of the parameters were examined.

### Decitabine demethylation and RA treatment

Decitabine/DAC (5-aza-2′-deoxycytidine; Sigma-Aldrich) is the most widely used inhibitor of DNA methylation [42]. It was firstly diluted with culture medium to 1μM to treat LN18, LN428 and U251 cells for 3 days by daily renewing decitabine-containing medium. Then the DAC-treated cells were further treated by 10μM RA for another 2 days, The effects of DAC and DAC combined RA on cell proliferation were determined by MTT, flow cytometry and coverslip-based TUNEL assay, morphological and ICC staining for CRABP-II and FABP5. RNA and protein samples were prepared from all groups for RT-PCR and Western blot analyses

### DNA sequencing of FABP5 RT-PCR product

Since FABP5 could be detected in U251 cells at RNA rather than protein level, DNA sequencing was conducted on the RT-PCR product generated from U251 cells to ascertain the specificity of PCR primers. Briefly, the PCR products in the length of 281 bp were amplified from 2.5 μl U251 RT products using the forward (5′-AGCAGCTGGAAGGAAGATGG-3′) and reverse (5′-CTGATGCTGAACCAATGCAC-3′) primers of FABP5. The PCR products were prepared for sequencing (3730xl sequenator, Life Tech, Texas, USA) according to the protocol provided with the BigDye Terminator Kit (Life Tech, Texas, USA). Sanger-sequenced clones of full-length PCR products were analyzed with Sequencing Analysis software (Version 5.2), then blasted with the sequence of the corresponding region in Reference mRNA of FABP5 (http://www.ncbi.nlm.nih.gov/gene/2171report=gene_table).

### Cell treatment with FABP5 inhibitor BMS309403

FABP5 competitive inhibitor BMS309403 (Santa Cruz) [28, 29] was dissolved in DMSO and diluted to the final concentration of 25μM with culture medium. Four experimental groups were set as follows: Group1, normal culture; Group 2, treatment with 1.2‰ DMSO as background control; Group 3, treatment with 10μM RA; Group 4, 25μM BMS309403 treatment for 72 hours; Groups 5, 25μM BMS309403 pretreatment for 6 hours followed by 10μM RA for 72 hours. The influence of BMS309403 in retinoic acid sensitivities of LN18, LN428 and U251 cells were determined by cell counting, viable/unviable cell discrimination (Automated Cell Counter, Bio-Rad, Singapore) and coverslip-based morphological staining.

### Statistical analysis

The results of cell counting and MTT data were evaluated with the independent-samples t-test and ANOVA. Data were presented as mean ± standard deviation (SD) of separate experiments (n≥10). When required, *p*-values are stated in the figure legends.
